# Ruminant microbiome data are skewed and unFAIR, undermining their usefulness for sustainable production improvement

**DOI:** 10.1186/s42523-024-00348-x

**Published:** 2024-10-25

**Authors:** Abimael Ortiz-Chura, Milka Popova, Diego P. Morgavi

**Affiliations:** Université Clermont Auvergne, INRAE, VetAgro Sup, UMR 1213 Herbivores Unit, Saint-Gènes-Champanelle, France

**Keywords:** Ruminant microbiome, Metadata, Global representativeness, Metagenome, Ontology

## Abstract

The ruminant microbiome plays a key role in the health, feed utilization and environmental impact of ruminant production systems. Microbiome research provides insights to reduce the environmental footprint and improve meat and milk production from ruminants. However, the microbiome composition depends on the ruminant species, habitat and diet, highlighting the importance of having a good representation of ruminant microbiomes in their local environment to translate research findings into beneficial approaches. This information is currently lacking. In this study, we examined the metadata of farmed ruminant microbiome studies to determine global representativeness and summarized information by ruminant species, geographic location, body site, and host information. We accessed data from the International Nucleotide Sequence Database Collaboration via the National Center for Biotechnology Information database. We retrieved 47,628 sample metadata, with cattle accounting for more than two-thirds of the samples. In contrast, goats, which have a similar global population to cattle, were underrepresented with less than 4% of the total samples. Most samples originated in Western Europe, North America, Australasia and China but countries with large ruminant populations in South America, Africa, Asia, and Eastern Europe were underrepresented. Microbiomes from the gastrointestinal tract were the most frequently studied, comprising about 87% of all samples. Additionally, the number of samples from other body sites such as the respiratory tract, milk, skin, reproductive tract, and fetal tissue, has markedly increased over the past decade. More than 40% of the samples lacked basic information and many were retrieved from generic taxonomic classifications where the ruminant species was manually recovered. The lack of basic information such as age, breed or sex can limit the reusability of the data for further analysis and follow-up studies. This requires correct taxonomic assignment of the ruminant host and basic metadata information using accepted ontologies adapted to host-associated microbiomes. Repositories should require this information as a condition of acceptance. The results of this survey highlight the need to encourage studies of the ruminant microbiome from underrepresented ruminant species and countries worldwide. This shortfall in information poses a challenge for the development of microbiome-based strategies to meet sustainability requirements, particularly in areas with expanding livestock production systems.

**Supplementary Information:**

The online version contains supplementary material available at 10.1186/s42523-024-00348-x.

## Background

The ruminant livestock sector is central to global food security and human nutrition. According to FAO [1], 17% of calories and 33% of the protein consumed in the world come from animal sources, and a large proportion of these come from ruminants. Likewise, ruminants improve the livelihoods and food security of millions of smallholders [2]. Compared to 2021, global demand for meat and milk is projected to increase by about 15% by 2031 [3]. The higher milk and meat production are projected to come largely from global expansion of cattle herds mostly in Africa (+ 13%), Latin America (+ 5%), and India (+ 3%), which are already home to the largest concentration of ruminants. This would lead to potentially adverse environmental consequences, such as greater greenhouse gas emissions, changes in land use, and negative effects on water use and quality [4].

To improve the sustainability of the ruminant livestock sector, rather than to increase herd size it is necessary to improve productivity (especially in regions with low productivity) by improving feed efficiency while preserving animal health and mitigating the environmental impact of production [5]. However, these productive traits vary widely around the world and depend on many factors including production system, animal genetics, husbandry practices, pasture and forage quality, and the use of feed supplements [6]. Additionally, the genetic potential of indigenous ruminant species and breeds would also help to address some of these major challenges, particularly in dry and tropical regions in developing countries, where population growth is expected to be higher [3, 7, 8].

Microbiomes associated with host animals are essential for the adaptation of the holobiont “the host and associated microbes”, to the environment [9] and there is growing interest in ruminant microbiomes. Fueled by recent advances in amplicon sequencing, metagenomics, metabolomics, and other omics technologies [10], there is a better understanding of the key role of the microbiome in ruminant health [11], performance [12] and environmental impact [13].

Globally, ruminant production is dominated by animals grazing and browsing native plants in natural ecosystems (rangelands), which cover 36% of the world’s land area, mostly in arid areas unsuitable for crop production [14]. Furthermore, each region or country has distinctive singularities because the type, quality, and quantity of rangelands are widely variable around the world [15]. Similarly, feeding management and diet, including the presence of plant secondary compounds, potentially influence the rumen microbial ecosystem and affect animal performance and health. For example, in tropical and subtropical regions of Latin America and the Caribbean, Africa, Asia, and northern Australia, *Leucaena leucocephala* is used as forage for cattle, but its secondary metabolites tannins and mimosine have antimethanogenic and toxic effects [16, 17]. Nevertheless, ruminants can develop adaptive microbial mechanisms to neutralize the toxic effects of plant secondary metabolites, thereby developing a gradual tolerance to these compounds in feedstuffs [18]. This singularity is naturally observed in extensive farming systems with adapted native livestock.

Ruminant microbiomes differ among species [19], breeds [20], and body sites [21], and they also differ among geographic locations as the feeding and husbandry conditions are different, as described above. To enhance our understanding of the functions, diversity, and interactions of the microbiome with the host, a robust and global reference genomic database representing all these situations is needed. The issue of representativeness has been addressed by projects such as the Global Rumen Census [22], and the Hungate1000 [23]. Although the Hungate collection represents a global effort, limitations remain because 93% of microbial cultures come from traditional livestock (cattle, sheep, and goats) that originated predominantly from developed countries (91%). Additionally, more recently, efforts to expand the database with culture-free metagenome-assembled genomes have been reported in Europe with local cattle [24] and in Africa with indigenous cattle [25]. In addition to species and breeds, other factors such as climate and available feed resources drive the microbial levers that can be used to meet societal and environmental demands. The aim of this work was to assess the suitability of existing microbial data for addressing sustainability criteria in areas of increasing livestock production. In view of the inherent constraints of these world regions – namely, their low and medium development status and the challenges posed by climate – we sought to ascertain the viability of microbial strategies developed in high-income countries for the reduction of livestock-related environmental impacts in such contexts. A second objective was to assess the extent to which ruminant microbiome data, in particular the metadata associated with samples, complies to Open Science and FAIR (findable, accessible, interoperable, and reusable) principles [26]. This is essential to improve reproducibility and transparency in scientific research [27], particularly in the field of microbiome research [28]. It is imperative that metadata standards are developed and made mandatory for data submission. This is a precondition for comparing studies and data generated by different technologies, which will result in increased accuracy in microbiome research. In addition, there is a rapid development of artificial intelligence approaches, particularly machine learning. The lack of thorough and consistent metadata will impede the assessment and prediction of microbiome-related phenotypes in re-analyses and meta-analyses of data. Other studies have reported on FAIR data issues in human [29] and agricultural [30] microbiome research. To date, however, there are no reports on the quality of ruminant microbiome metadata. Nor is it known how globally representative it is, or which ruminant species and body sites are most commonly studied. To address these information gaps, we explored and summarized information on the ruminant microbiome research metadata according to animal species, geographic location, body site, information about age, sex, and breed of the host, and system of production using databases from the International Nucleotide Sequence Database Collaboration. We also compared the country of origin of samples with the ruminant population as a proxy to assess the representativeness of regional production systems.

## Methods

### Data search and processing

This study was performed following the Preferred Reporting Items for Systematic Reviews and Meta-Analyses (PRISMA) guidelines [31]. We focused on the ten most important farmed ruminant species: Cattle (*Bos taurus*), sheep (*Ovis aries*), goat (*Capra hircus*), yak (*Bos grunniens*), buffalo (*Bubalus bubalis*), bison (*Bison bison*), and the Old World (*Camelus dromedarius* and *Camelus bactrianus*) and New World (*Lama glama* and *Vicugna pacos*) camelids. Metadata available for these ten species were exported using the search query “txid[Organism] AND biosample sra[filter] AND “public“[filter]” in the NCBI BioSample database (https://www.ncbi.nlm.nih.gov/biosample), accessed 28 July 2022. For instance, using the search category “bovine gut metagenome” in the NCBI taxonomy browser (https://www.ncbi.nlm.nih.gov/Taxonomy/Browser/wwwtax.cgi), accessed 28 July 2022, we obtained the taxonomy identifier for the search field “txid506599 [organism]”. Then, the search query requests all samples classified in this search category. This procedure was repeated for each ruminant species combining or not with body site (gut, oral, skin, vaginal, lung, nasopharyngeal, feces, reproductive system, blood, milk, urinary tract, tracheal, eye and semen) and the word metagenome (e.g., sheep gut metagenome).

Following the initial search, we found only three search categories available in the NCBI taxonomy. However, we found other generic categories nested under “gut metagenome” and “metagenome” that were not explicitly labelled as cattle, sheep or goats but contained many ruminant related samples (Table 1). Sample identifiers and all associated tags were loaded into a full XML file format. The XML files were converted into a single data frame format using the XML and xml2 packages in R software version 4.2.0 [32], which allowed extracting the information in the principal nodes (publication date, submission date, id, project name, and attributes), and in the sub-nodes of the attribute node (host, geolocation and source of the sample, among others).

**Table 1:** Search categories available in the NCBI taxonomy and search query in the NCBI BioSample database

Ruminant species	Search category	Taxonomy	Search query
Cattle	Bovine gut metagenome	txid506599	txid506599[Organism] AND biosample sra[filter] AND “public“[filter]
	Bovine metagenome	txid1218275	txid1218275[Organism] AND biosample sra[filter] AND “public“[filter]
Sheep	Sheep gut metagenome	txid1904483	txid1904483[Organism] AND biosample sra[filter] AND “public“[filter]
	Sheep metagenome	txid1898966	txid1898966[Organism] AND biosample sra[filter] AND “public“[ filter]
Goats	Goat gut metagenome	txid2809082	txid2809082[Organism] AND biosample sra[filter] AND “public“[filter]
Generic	Gut metagenome	txid749906	txid749906[Organism] AND biosample sra[filter] AND “public“[filter]
Generic	Metagenome	txid256318	txid256318[Organism] AND biosample sra[filter] AND “public“[filter]

Data from the two generic categories were analyzed to find samples associated with the ten ruminant species. For this, we manually checked the “host” attribute and, if it was empty, we checked the rest of the attributes and added any information explicitly indicating that the sample was from one of the ruminants of interest for this study. For buffalo, yak, bison, and all camelids’ species metadata were only retrieved from the generic search categories, as we did not find any specific taxonomy identifier associated with search categories. For cattle, sheep, and goats, a total of 5,567, 2,607, and 1,656 samples, respectively, were retrieved from generic search categories and included in the analysis.

Prior to the final sample count for each ruminant species, we filtered out those samples that were from the environment (e.g., soil, drinking water, air, cages), associated with animal samples that were processed industrially (e.g., cheese) or included in the experimental design but not obtained from the ruminant animal (e.g., negative control and mock). Therefore, we considered only samples coming directly from the animal’s body. The result is a dataset containing 47,628 sample metadata from multiple body sites.

In analyzing the data, we found that a large proportion of the samples lacked basic information about the host attributes, such as age, sex, and breed. To retrieve this information, we reverified the metadata according to the sample and bioproject identifier in the NCBI database. If the information was not found in the bioproject description, we performed a literature search to find metadata associated with the bioproject identifier linked to the samples. Due to the high heterogeneity of the data, we recategorized some attributes to render the information contained in the dataset more meaningful. The age of cattle was categorized into calves (birth to 1 year), yearlings (> 1 to 2 years), and adults (> 2 years); for sheep and goats, lamb or kid (birth to 5 months), yearlings (> 5 months to 1 year) and adults (> 1 year). Although there is no specific attribute for in vivo or in vitro samples in the metadata set, we were able to separate in vitro from in vivo samples by manually searching for those samples associated with reactor, culture, in vitro, and RUSITEC. Likewise, in the cattle metadata, we added the attribute production system, associating it directly with the breed, e.g., breed specialized in milk production such as Holstein, so it was assigned to the dairy production system. Finally, the sequencing technique employed was not explicitly described in the available attributes of each sample, although a few had tags referring to 16S rRNA gene and shotgun metagenomics. Therefore, this information was not taken into account in this study.

## Descriptive analysis and representative proportion

For the general descriptive analysis and for each ruminant species, we created the pivot table of the Excel file considering the attributes, biosample ID, ruminant species, date, body site (categories: oral [subcategory: oral, tonsil and saliva], gut [esophageal, rumen, reticulum, omasum, abomasum, duodenum, jejunum, ileum, cecum, colon, rectum, and anus], feces [feces], respiratory system [nasal, lung, larynx and trachea], milk [milk and colostrum], fetal tissue [liver, placenta, kidney, ileum, amniotic fluid, cecum, meconium, allantoic fluid, rumen, fetal gut, and umbilical cord], skin [skin, foot, udder skin, and ventral skin], reproductive system [uterus, vagina, and penis], liver [liver], mammary gland [udder and teat], blood [blood], eye [eye], musculoskeletal system [muscle and joint] and ears [ears]), sample type, country, breed, sex, age, and production system (for cattle only) (10.57745/KH3WRF). The bar, alluvial, and donut charts were generated with the ggplot2 package [33] using R Software.

Cattle and sheep were the only species considered to estimate the patterns of over- or underrepresentation by country in relation to its global cattle and sheep population, because they were the species with the highest number of samples (~ 90%). For this purpose, we downloaded the total population of cattle and sheep per country for 2020 using the FAOSTAT database [34] (https://www.fao.org/faostat/fr/#data/QI), accessed 26 October 2022. Consequently, the representation index was estimated with data from the country’s share of the world population (of cattle or sheep) and the country’s share of the microbiome samples following the methodology of Abdill et al. [29]. Briefly, for countries with a percentage of samples greater than the percentage of cattle or sheep populations, we divided the former by the latter to obtain a number indicating how many times more samples are present than expected. For countries where the percentage of samples was less than the percentage of cattle or sheep populations, we took the negative reciprocal of this number. The provisional result leaves overrepresented countries with positive scores and underrepresented countries with negative scores. After removing scores for countries with fewer than ten samples, we scaled the positive scores to be between 0 and 100 and separately scaled the negative scores to be between 0 and − 100. The R package maps and ggplot2 were used to graphically display the representativeness maps. To add more variation to the color coding of countries, the scaled representativity indices were transformed to log_10_.

## Results and discussion

### Global sample metadata distribution of ruminant microbiome samples

**Fig. 1:**
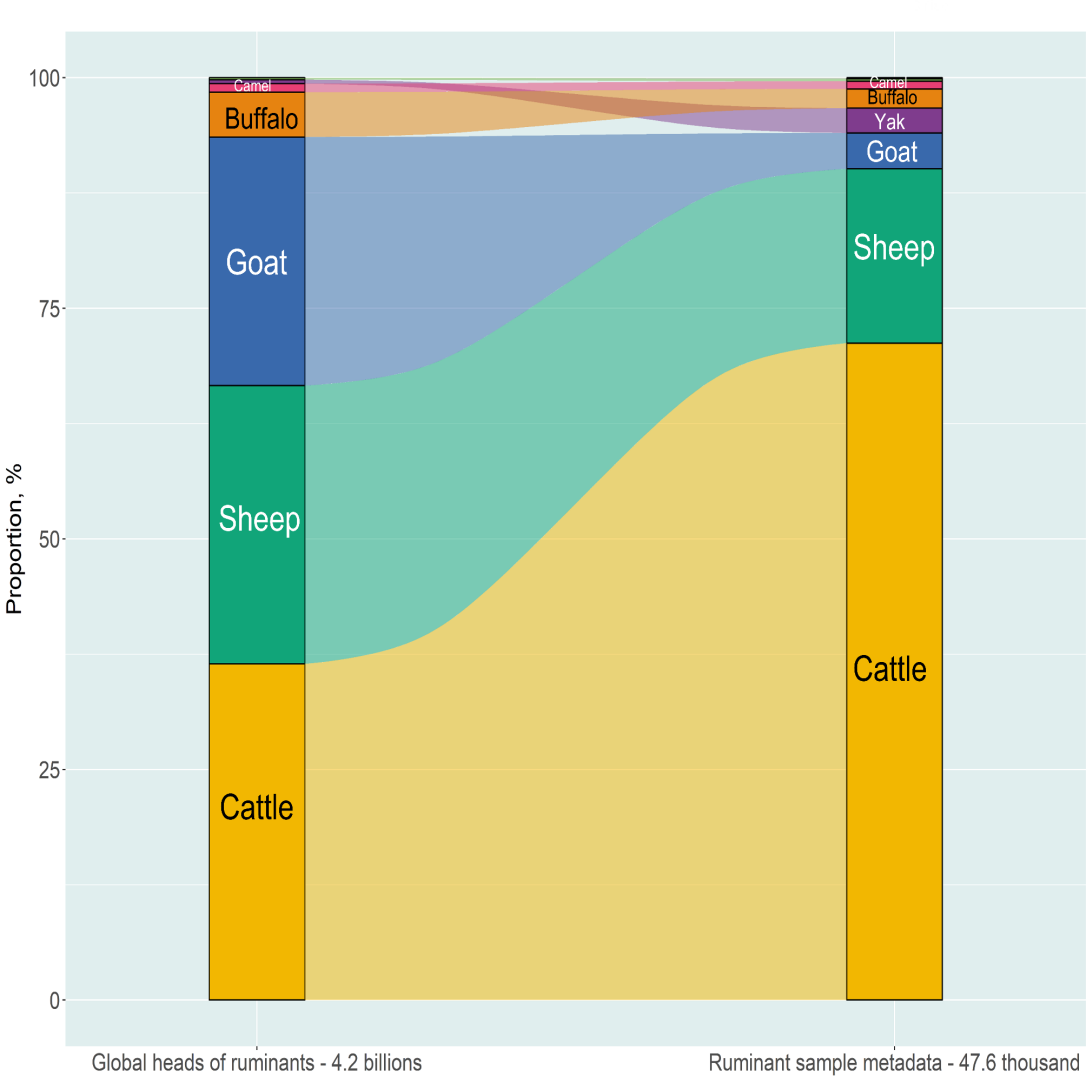
Comparison of proportion (%) between worldwide heads and sample metadata of ruminant species.

A dataset with 47,628 sample metadata was obtained from ten farmed ruminant species (Fig. 1). Cattle (including *Bos taurus* and *Bos indicus*) represented 71.2% of the samples followed by sheep at 18.9%. Other species were goat (3.9%), yak (2.7%), and buffalo (2.1%). The rest of the samples (~ 1.2%) are from four camelid species and from bison. Samples from live animals were dominant compared to those from in vitro experiments (93.5% vs. 6.5%, respectively). Present estimates of the worldwide farmed ruminant population are about 4.2 × 10^9^ heads, including yak [35] and bison [36] populations that are not counted in FAOSTAT [34]. Cattle (36.45%), sheep (30.17%) and goats (26.95%) account for the largest populations, followed by buffalo (4.86%), camelids (0.92% Old World and 0.21% New World), yak (0.42%) and bison (0.01%). A comparison between the proportion of samples and head numbers for each ruminant species to identify gaps in the global research effort in regards to some ruminant populations related to others is prone to criticism. Factors such as economic and regional importance should be considered for a finer interpretation. The use of head numbers or livestock units [37] will also modify the results. Nevertheless, samples from sheep, goats and buffalo seem clearly underrepresented. This is even more evident considering that these three species are particularly abundant and economically important in African and Asian countries [38], which have a low overall contribution of samples (see below).

Geographic location was a frequent metadata attribute that allowed us to identify the country of origin of the sample. We identified a total of 52 countries with China, the USA and Canada contributing more than half of the samples. Other countries contributing more than 1% of the samples were nine European countries, New Zealand, Australia, Israel, Brazil, and Japan. The remaining 31 countries contributed 5.6% of the samples (Supplementary Table 1).

**Fig. 2:**
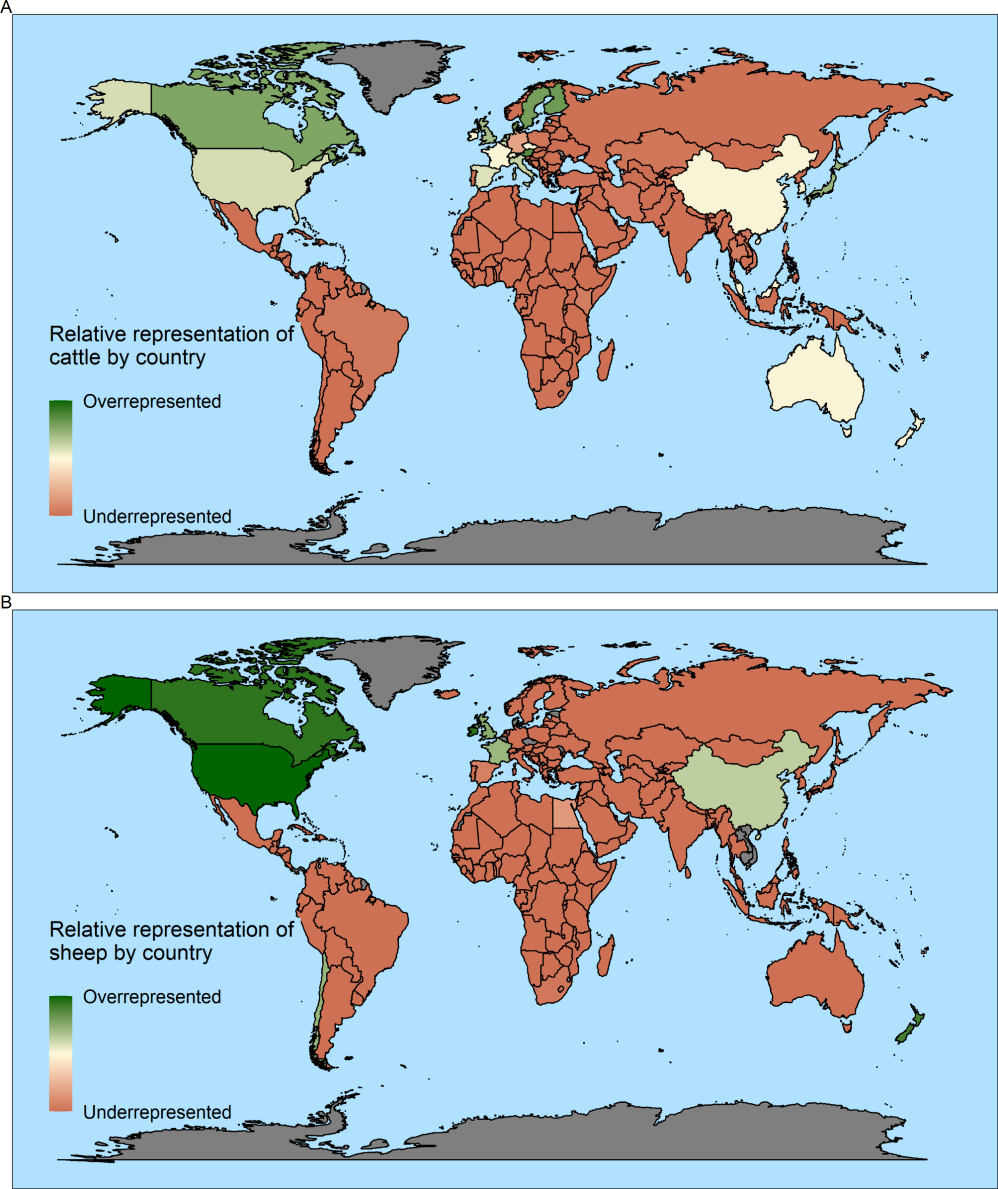
Data from cattle (**A**) and sheep (**A**) associated microbiome relative to abundance of livestock population in the world. Green hues mark countries where microbiome samples are overrepresented relative to their cattle or sheep populations, and red hues mark countries that are underrepresented or that have no sample metadata. Countries with no data on cattle or sheep populations in the FAOSTAT database (accessed 26 October2022) are marked in gray

For a better understanding of the microbiome metadata representation and given that cattle and sheep represent about 90% of the total samples, we analyzed the data separately for each of these two species. We then used the geographic location attribute, and along with information on cattle populations in countries worldwide, we evaluated the representativeness of sampling efforts on a global scale. To obtain a clear picture, we filtered the dataset by removing in vitro samples and countries that had a low number of samples (< 10). The latest available data for the worldwide cattle population is 1.53 × 10^9^ heads [34]. One animal out of four in the world is from only two countries, Brazil and India. Other countries with large cattle populations are the USA (6.1%), Ethiopia (4.3%), China (3.9%), Argentina (3.5%), Pakistan (3.4%), Mexico (2.4%), Chad (2.2%) and Sudan (2.1%). However, the samples mainly originated from the USA (25.4%), Canada (13.2%), China (12.1%), Austria (6.5%), the UK (5.9%), and Israel (5.1%) (Supplementary Fig. 1). Countries with a low to moderate cattle population, for example, Israel, Austria, Denmark, Finland, Sweden, Canada, Japan, and the UK, were overrepresented. In contrast, out of the 25 countries with the largest cattle populations, 21 are underrepresented (Fig. 2A). Furthermore, out of the 190 countries reported with cattle populations, 144 have zero samples reported in this database.

As for cattle, the geographic location and information for the worldwide sheep population were analyzed. Our results showed that, although the sheep population from the USA, Canada, New Zealand, and Ireland did not exceed 3% of the total, 55% of the sheep microbiome samples originated from these four countries. Consequently, these countries were overrepresented (Fig. 2B). China has the largest sheep population in the world (13.7%) and accounts for 32.3% of the total samples; hence, as well as the UK and France, they are considered well-represented countries. In contrast, 7 of the top ten countries in sheep populations (not including China, India, or the UK) did not register any samples (Supplementary Fig. 2). Likewise, India, Brazil, South Africa, Spain, and Egypt were ranked as the most underrepresented countries. Remarkably, out of the 173 countries reported with sheep populations, 127 have zero samples reported in this database.

## Sample metadata information from the three most abundant ruminant species

Regarding the body site of origin, the vast majority of samples (~ 87%) come from the gut, particularly from rumen, that represented 56% of the total, and feces, and were prevalent in all ten ruminant species. Other body sites and biological matrices represented about 13% of the samples. These are in decreasing order of importance from respiratory system, milk, fetal tissue, skin, and reproductive system categories (Table 2). Samples from body sites other than the gut and feces were mainly found in cattle and sheep. Minor categories represented less than 1% of the total samples (listed in Supplementary Table 2).

**Table 2:** Sample metadata distribution by body site and ruminant species ^1^

Items	Sample counts	%	Ruminant species
Gut	30,452	63.9	C, S, G, Y, Bu, BC, DC, A, LL and Bi
Esophageal	5		C
Rumen	26,652		C, S, G, Y, Bu, BC, DC, A, LL and Bi
Reticulum	131		C, S, G, Y and Bu
Omasum	150		C, S, G, Y and Bu
Abomasum	252		C, S, G, Y, Bu and BC
Duodenum	374		C, S, G, Y, Bu and A
Jejunum	567		C, S, G, Y, Bu and A
Ileum	496		C, S, G, Y, Bu, BC and A
Cecum	405		C, S, G, Y, Bu and A
Colon	658		C, S, G, Y, and Bu
Rectum	525		C, S, G, Y, Bu and DC
Anus	73		S and G
Gut^2^	164		C, BC, and DC
Feces	10,825	22.7	C, S, G, Y, Bu, BC, DC, A, LL and Bi
Respiratory system	1759	3.7	C, S, Y and DC
Milk	1389	2.9	C, S and Bu
Fetal tissue	1001	2.1	C and S
Skin	752	1.6	C and S
Reproductive system	624	1.3	C and S

^1^ C = Cattle, S = Sheep, G = Goat, Y = Yak, Bu = Buffalo, BC = Bactrian camel, DC = Dromedary camel, A = Alpaca, LL = Llama, and Bi = Bison.

^2^ Sample metadata tagged as gut.

**Fig. 3:**
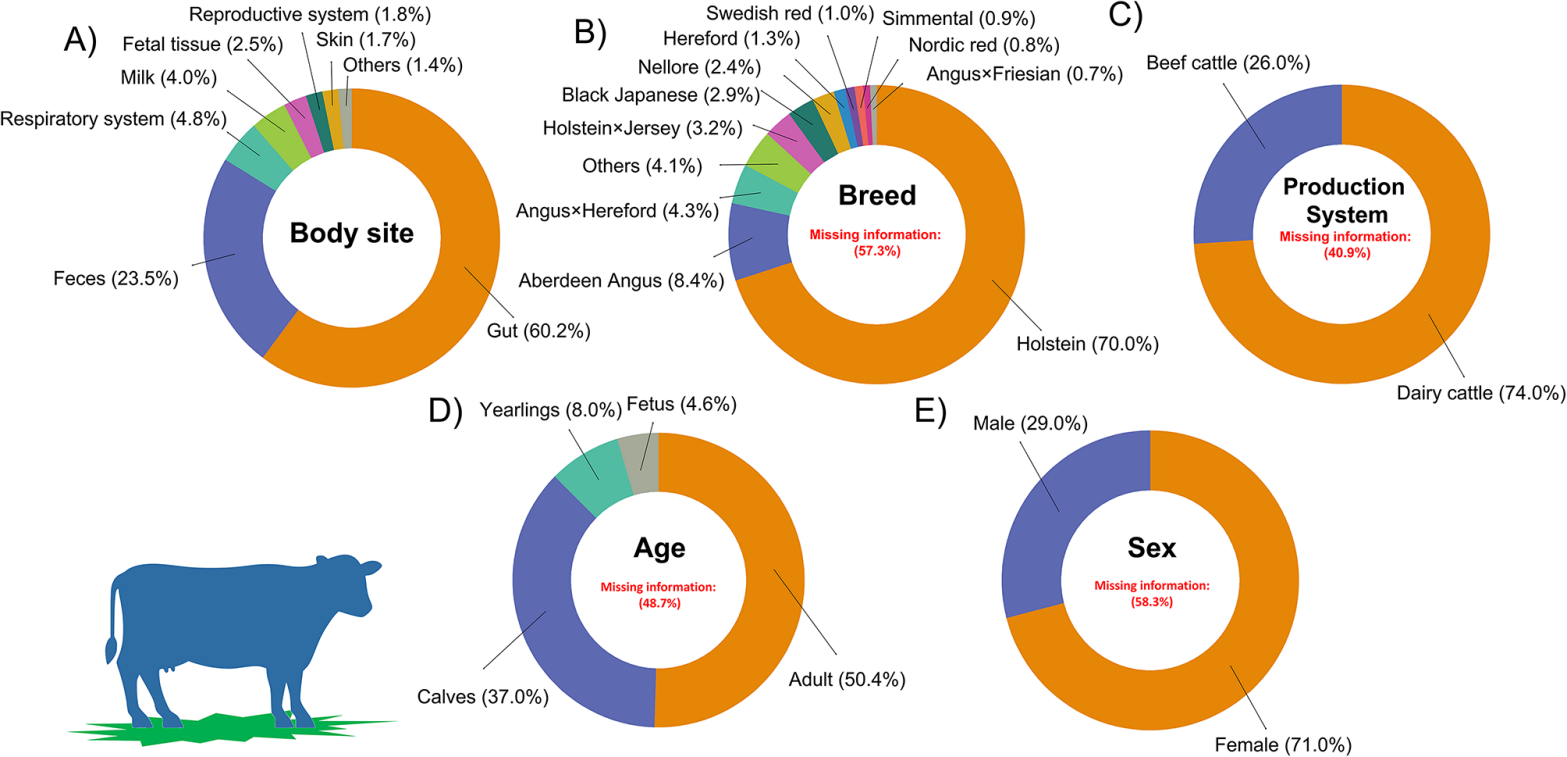
Cattle sample distribution according to five different categories: body site (**A**), breed (**B**), production system (**C**), age (**D**), and sex (**E**). For the body site and breed categories, body sites and breeds with less than 1% and 0.3% representation, respectively, were grouped in the subcategory others. Missing information on breed, production system, age and sex were not included as subcategories in the figures

Cattle represented 71% of all sample metadata, and the body site was the attribute where the information was most complete. However, the information was not straightforward, and it was only recovered after refining the search on the attribute “description” of the bioproject or by manually searching the associated publications. We found 13 categories for the body site attribute. The categories Gut and Feces were also dominant, representing about 8 out of 10 samples (Fig. 3A). Other relevant categories were: respiratory system, fetal tissue, milk, reproductive system, skin, liver, oral, mammary gland, blood, eye and musculoskeletal system (Supplementary Table 3). The breed is an important descriptive information in any animal study but it was not reported in the majority of sample metadata (57.3%). In spite of the limited availability of breed attribute data, Holstein was the dominant breed (70.0%), followed by Aberdeen Angus, Angus × Hereford crossbreed, Holstein × Jersey crossbreed, and Black Japanese (which refers mainly to the Wagyu breed) (Fig. 3B). Similar to breed, fundamental attributes for reusability and reinterpretation of sequencing data such as production system, age, and sex were poorly completed. No information on these attributes was found in 40 to 58% of the samples. The available data should be interpreted with caution but there is a predominance of sample metadata from dairy versus meat production systems (74% vs. 26%, respectively) (Fig. 3C), which is opposite to the global cattle structure, 17% for dairy cattle and 83% for beef cattle [34, 39]. Furthermore, samples from adult animals are higher than those from calves but otherwise they can be considered equilibrated (Fig. 3D). Whereas, the female category (Fig. 3E) is more abundant than the male category, which is expected given the sex ratio in commercial cattle herds.

**Fig. 4:**
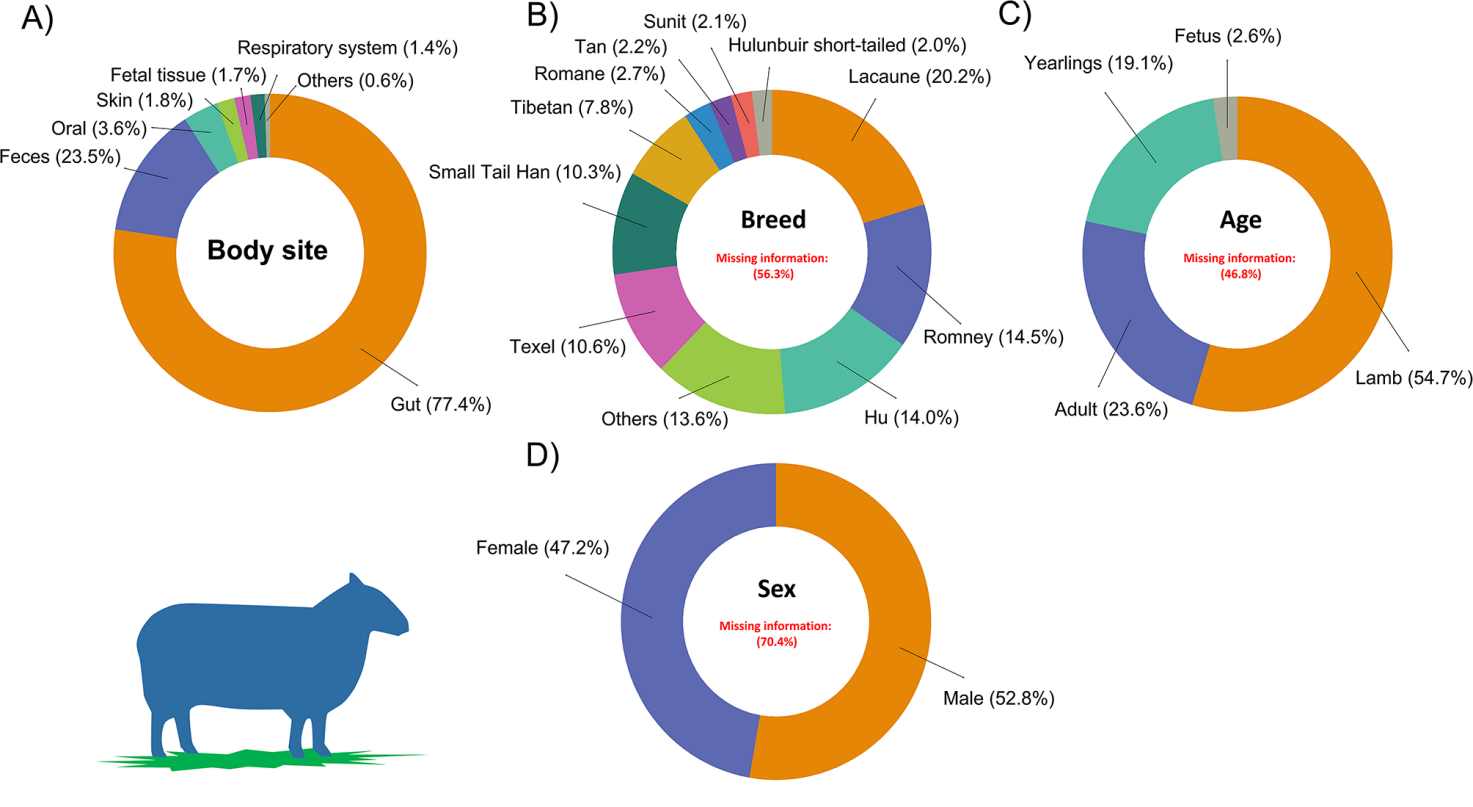
Sheep sample distribution according to four different categories: body site (**A**), breed (**B**), age (**C**) and sex (**D**). For the body site and breed categories, body sites and breeds with less than 1% and 2%, representation, respectively, were grouped in the subcategory others. Missing information on breed, age and sex were not included as subcategories in the figures

For sheep, a total of 9,003 sample metadata were found. As in cattle, the gut and feces categories of the body site predominated (90.9%) over the other categories (Fig. 4A) (Supplementary Table 3). Likewise, for the breed attribute, there was a high percentage of missing data (56.3%). We found a total of 31 breeds, and the most abundant were the Lacaune (20.2%), Romney (14.5%), and Hu sheep (14.0%) breeds. Most breeds were poorly represented (< 1%) (Fig. 4B). Finally, for the attributes age and sex, although there was a high percentage of samples with missing data, lambs and adults were the most represented categories (Fig. 4C), and similar proportions were observed for males and females (Fig. 4D).

Goat results showed only two body site categories, gut and feces (Supplementary Table 3). Although 29 breeds were identified, about 50% of the samples lacked this attribute (Supplementary Table 4). The predominant breeds were: Liuyang black, Boer, Black fattening, and Xiangdong black. Approximately half of the breeds that were informed in the metadata have a Chinese origin, as 90% of the samples originated from China (Supplementary Table 5). Seventeen other countries registered samples, but they represented less than 10%. We found no or few samples from countries with large populations of goats (e.g., India, Nigeria, Pakistan, Bangladesh, and Ethiopia). Finally, although the kids and female categories predominated in the age and sex attributes, respectively, there was a higher percentage of missing data (45.5 to 67.8%) (Supplementary Tables 6 and 7).

### Sample metadata information from minor ruminant species

Outside of the major ruminant species, the number of total samples from other ruminants (yak, buffalo, camel, camelid, and bison) were equilibrated compared to their worldwide population (~ 6%). Regardless of the ruminant species, the gut and feces categories were the most prevalent among these seven ruminant species (Supplementary Table 8). Likewise, some respiratory system and milk samples were reported from yaks, camels, and buffaloes. Sample metadata originated mainly from the Asian continent (91%). China and India had the largest number of samples (Supplementary Table 9); China was highlighted by the number of samples of yak (1,280 samples), and both countries contributed 916 samples of buffalo. For the Dromedary camel, India, Egypt, Iran, and other countries contributed 151, 108, 44, and 11 samples, respectively. There were 79 samples from Bactrian camels originating from Russia, China, Italy, and Denmark. Likewise, for bison, 58 samples were reported from the USA, Canada, and Mexico. It is noted that for New World camelids most samples were from outside the main geographic area of production and origin. There were 123 alpaca samples from the USA and New Zealand, and only eight llama samples, six from Argentina and two from France.

## Database representation and FAIR principles

**Fig. 5:**
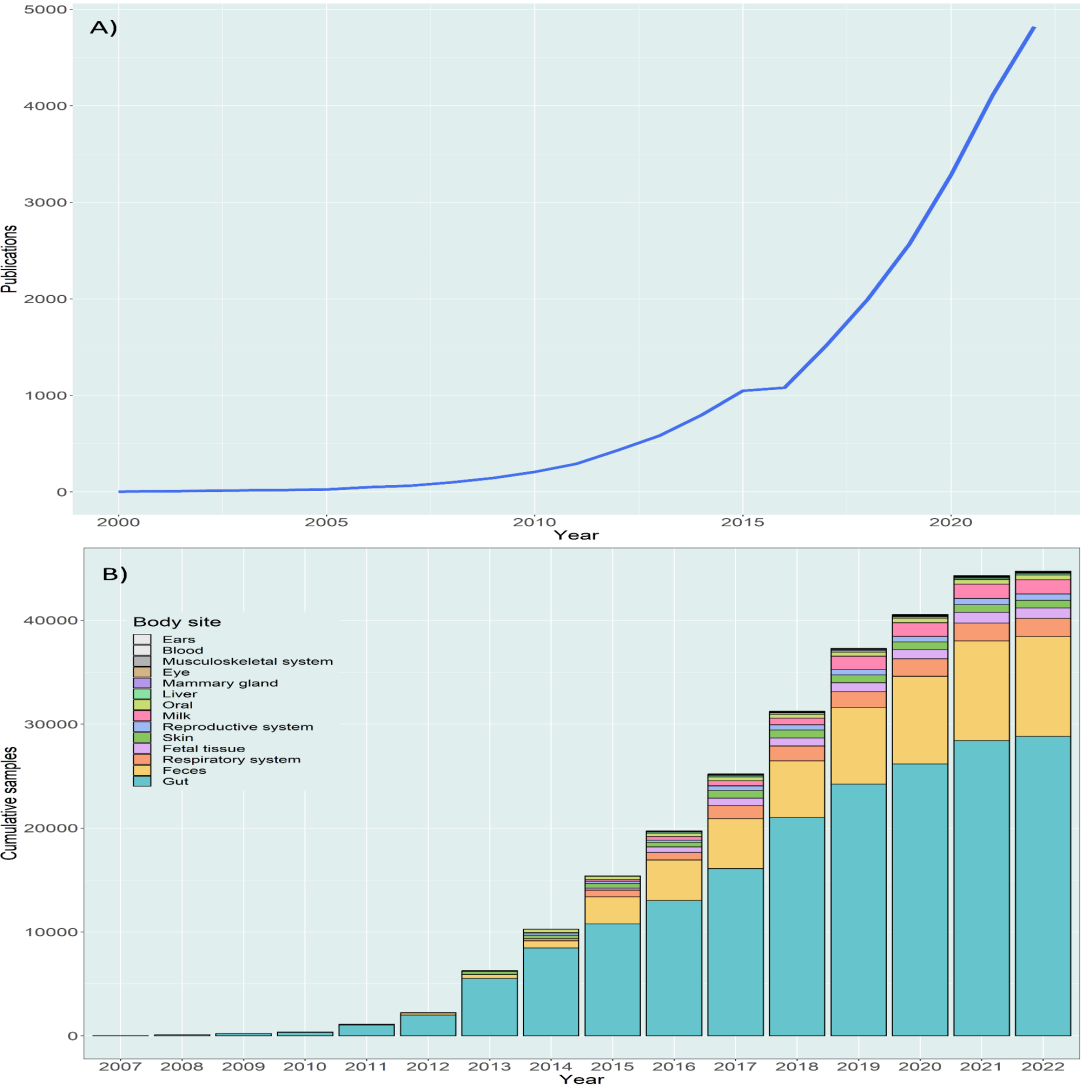
Timeline evolution of the ruminant microbiome studies. (**A**) Cumulative number of published papers related to ruminant microbiome (PubMed search query: microbiome OR microbiota OR metagenome AND cow OR cattle OR sheep OR lamb OR rumen OR ruminants OR camels OR camelids OR Buffalo OR Bison [cumulative total = 4,820]) from 2000 to 2022 (up to 26 October). (**B**) Cumulative evolution of total sample metadata by body site attribute. Bar chart plots were made using body site data of cattle, sheep and goats, including in vivo and in vitro samples. Metadata for 2022 is up to June

Our results, based on the number of scientific papers (Fig. 5A) and sample metadata evolution (Fig. 5B), suggest a growing interest in ruminant microbiome studies with the aim of understanding the function of the holobiont organism and its linkages with animal health, production efficiency, and environmental impact [11, 13]. Additionally, advances, and cost reductions, in high-throughput sequencing technologies have contributed to the increased data volume in the last decade [10]. The results indicate that the GIT is the most studied body site in farmed ruminants (Supplementary Fig. 3). This is explained by the importance of the GIT microbiota to the major challenges facing ruminant production, namely reducing greenhouse gas emissions, increasing feed efficiency, and preserving animal health [40–42]. In addition, the number of samples from the respiratory tract, milk, skin, reproductive tract, and fetal tissue has increased exponentially over the past decade, reflecting the increased interest in better understanding how resident microbiota are associated with health problems, such as mastitis [43], lameness [44] and respiratory disease [45].

The quality and depth of the microbiome data from farmed ruminants is steadily improving, allowing us to explore their connection to essential biological processes relevant to production and health. Several projects and international initiatives e.g [22, 23]. are contributing data, expanding the ruminant microbiome. However, the existing metadata and samples mainly originated from production systems prevalent in high-income countries, and there is still a large number of regions with large ruminant populations where metadata were scarce or nonexistent, e.g., countries from South America and the Caribbean, Western Asia, Eastern Europe, and the African continent.

It is, therefore, urgent to rethink and encourage ruminant microbiome studies in underrepresented countries worldwide. It is imperative to obtain information from indigenous breeds and less represented ruminants reared under harsh environmental conditions from low- and middle-income countries where they contribute to food security [7]. These regions are where ruminant populations are increasing and where ruminants contribute the most to the economic and environmental sustainability (adaptation and mitigation to climate change) of local human populations. We also consider that the vast but underexplored genetic diversity of ruminant microbiomes could be mined for the discovery of new genes and potentially valuable new microbial products for the biotechnology industry [46]. Finally, a better understanding of pathogenic microbes and their interactions with other microbiomes in ruminants and their environment may contribute not only to the development of healthy and sustainable livestock, but also to improved public health following the “One Health” approach [47, 48].

A main result of this study was the poor quality of the available metadata. For instance, there was no global consensus for the taxonomic assignment of the sample metadata to a ruminant species since much of the data were manually retrieved from generic taxonomies such as metagenome or gut metagenome, which include the vast majority of animal species. Likewise, we found samples of sheep and yak in the bovine metagenome and bovine gut metagenome taxonomies. All of this made it much more difficult to find and retrieve metadata. A further issue when refining the metadata information was the difficulty of distinguishing the nature of the samples. For instance, samples from in vitro studies were difficult to distinguish from in vivo because these were not explicitly defined in the metadata. Therefore, we classified samples as in vitro when they were associated with the reactor, culture, RUSITEC, or in vitro, and the remaining samples were considered in vivo. It is also important to know that in vitro anaerobic culture samples are taken from bottles or tubes, which often come from three or four individual animals or their mixture [49]. For this reason, it was important to exclude them from the proportional representativeness analysis as they do not truly represent a sample from an individual animal per se. An additional key point regarding data quality was incomplete (basic, but essential) host information. Although the associated bioprojects in the literature and those with more information on their attributes allowed us to complete basic host information, most of the samples did not have complete information on breed, sex, age, and production system, which was missing in more than 40% of samples. Therefore, our results related to host attributes, except for ruminant species, country, and body site, which did contain complete information, are partial and should be interpreted accounting for this caveat.

The completeness and standardization of metadata using a common language (ontology) are essential not only to ensure the quality of the available data, but also to ensure transparency, reproducibility, and reusability of data for secondary studies (meta-analyses and reviews, among others) [50]. To address these issues, there is a checklist with the minimum information about any (x) sequence (MIxS) required to be completed in the repositories [51], and international initiatives are underway to improve the quality of metadata, e.g., The National Microbiome Data Collaborative (NMDC) [52], the Genomic Standards Consortium (GSC) [53], and the Agricultural Microbiome Data [30]. However, we did not observe major progress, even in more recent studies, toward incorporating these recommendations into metadata information from ruminant microbiome research. Although some issues related to metadata quality could be related to legal concerns (e.g., intellectual property protection), we believe that the major drawback is the lack of a common ontology that correctly describes the host organism and that insufficient emphasis is placed on metadata as an indissociable element of the sequencing data to follow FAIR principles. Finding the correct ontology of animal-associated microbiomes to submit metadata is therefore a challenge to improve metadata quality. One possibility to facilitate the search for nonredundant ontology is to hierarchize the data structure for the ruminant microbiome, as was suggested for the plant-associated microbiome [50], and to adopt some categories of metadata (i.e., production system, productive and health traits, sampling method, processing and storage for host samples and sequenced materials) suggested in the checklist of the Agricultural Microbiome Data [30]. Host information on the (ruminant) species, breed, age, and sex are obvious basic information that should be a minimum prerequisite to deposit microbiome sequencing data. Furthermore, adopting and using livestock-specific ontologies that define animals in their environment, such as the Animal Trait Ontology of Livestock (www.atol-ontology.com), and others related to productive and health traits such as the Animal QTLdb database (https://www.animalgenome.org/QTLdb), would provide much-needed information for data reuse. Given that it is well known that the GIT microbiota is modulated primarily by the type and quality of the diet [54], further information on the type of diet and its possible associations with productive and health traits in the global microbiome database would be interesting. The animal research microbial community should improve its compliance with open data and FAIR principles that are required by international and national funding agencies. Training focused on quality standards, FAIR principles, and ontology for microbiome data could help promote adoption.

Limitations of this study may include duplicate or repeated values for some samples obtained from repeated measures studies or longitudinal studies. This type of information was not found in the list of attributes in the metadata, highlighting the need to improve the information collected as metadata. This is important given the growing interest in studying the long-term effects of dietary interventions on the gut microbiome and the development of the gut microbiome in early life in animals [55–57]; it is therefore likely that the number of samples from longitudinal studies will increase, and it is important that the nature of these samples should be clearly defined in the metadata. Our findings in this study are also limited to the databases of the International Nucleotide Sequence Database Collaboration [58], which includes the European Nucleotide Archive EMBL-EBI [59], the GenBank database of the NCBI [60] from the USA, and the DNA Data Bank of Japan [61]. Other databases, such as Metagenomics RAST (MG-RAST) [62], Genome Sequence Archive (GSA) [63], Global Catalogue of Metagenomics (gcMeta) [64], and Genomes Online Database (GOLD) [65], are likely to have different global representation patterns, although their orders of magnitude may be small and redundant. Furthermore, even using the same database, the results may differ from those found in our study. A study by Hu et al. [66], also using the International Nucleotide Sequence Database Collaboration archive, reported 3.6 times fewer samples from cattle. This suggests that there are “ruminant metagenome” samples incorrectly deposited under generic taxonomic identifiers, emphasizing the need to select the correct taxonomic identifier for samples when submitting data.

Microbiome-based solutions in ruminants have the potential to help address global environmental challenges, food security and antimicrobial resistance, while maintaining and promoting human, animal and ecosystems health, which is critical for achieving sustainability. However, these efforts may be ineffective if information on ruminant microbiomes is not widely available, as demonstrated in this study. Governmental and non-governmental organizations should take action to promote the study of ruminant microbiomes in underrepresented ruminant species and countries. Similarly, to achieve this global goal and to overcome the bottlenecks of microbiome research, i.e., reproducibility and replicability, the promotion of best practices for sharing of data, metadata, bioinformatics and statistical codes [27], and the adoption of Standard Operating Procedures (covering experimental design, sample collection and processing, and bioinformatics and statistical workflows) are emerging and necessary actions.

## Conclusions

We highlighted that certain ruminant species and geographical regions are underrepresented in the ruminant-associated microbiome dataset. This is an issue for the development of microbial strategies to meet sustainability challenges in areas with expanding livestock production systems, highly exposed to climate change and facing increased demand for high quality proteins. This study shows that incomplete metadata accompanies ruminant microbiome sequencing data in public repositories, hindering their reuse. This is an area where improvements can easily be made. The first step is to assign the correct taxonomic identification. Additional measures should ideally include the use of customized ontologies. These can be accessed from public repositories for metadata collection. As a condition of acceptance, repositories should require basic sample metadata information.

## Electronic supplementary material

Below is the link to the electronic supplementary material.

Supplementary Material 1

Supplementary Material 2

Supplementary Material 3

Supplementary Material 4

## Data Availability

The datasets generated and/or analyzed during the current study are available in the Data Gouv repository, 10.57745/KH3WRF.
