# Supplementary material for: Ruminant microbiome data are skewed and unFAIR, undermining their usefulness for sustainable production improvement
Source: Anim Microbiome. 2024 Oct 25;6:61. doi: 10.1186/s42523-024-00348-x (PMC11515148; doi:10.1186/s42523-024-00348-x)
Supplement: Supplementary file 1 — Supplementary Material 1 [file 42523_2024_348_MOESM1_ESM.doc]

**Supplementary Table 1**. Total sample metadata by country

| **Items** | **Sample counts** | **%** |
| --- | --- | --- |
| China | 10915 | 22.92 |
| USA | 9963 | 20.92 |
| Canada | 4597 | 9.65 |
| New Zealand | 3263 | 6.85 |
| UK | 2572 | 5.40 |
| Austria | 2189 | 4.60 |
| Israel | 1738 | 3.65 |
| France | 1445 | 3.03 |
| Brazil | 1277 | 2.68 |
| Denmark | 1041 | 2.19 |
| Japan | 1025 | 2.15 |
| Australia | 860 | 1.81 |
| Germany | 832 | 1.75 |
| Italy | 765 | 1.61 |
| Sweden | 692 | 1.45 |
| Spain | 598 | 1.26 |
| Netherlands | 524 | 1.10 |
| Ireland | 519 | 1.09 |
| Finland | 470 | 0.99 |
| India | 457 | 0.96 |
| South Korea | 269 | 0.56 |
| Belgium | 212 | 0.45 |
| Kenya | 174 | 0.37 |
| Iran | 152 | 0.32 |
| Egypt | 124 | 0.26 |
| Czech Republic | 103 | 0.22 |
| Uruguay | 93 | 0.20 |
| Russia | 91 | 0.19 |
| Switzerland | 91 | 0.19 |
| South Africa | 83 | 0.17 |
| Thailand | 82 | 0.17 |
| Turkey | 59 | 0.12 |
| Chile | 43 | 0.09 |
| Mexico | 39 | 0.08 |
| Kazakhstan | 37 | 0.08 |
| Viet Nam | 32 | 0.07 |
| Myanmar | 31 | 0.07 |
| Nigeria | 31 | 0.07 |
| Poland | 30 | 0.06 |
| Malaysia | 28 | 0.06 |
| Colombia | 24 | 0.05 |
| Saudi Arabia | 12 | 0.03 |
| Argentina | 10 | 0.02 |
| Slovenia | 10 | 0.02 |
| Serbia | 9 | 0.02 |
| Iceland | 4 | 0.01 |
| Norway | 4 | 0.01 |
| Hungary | 3 | 0.01 |
| Pakistan | 3 | 0.01 |
| Cuba | 1 | 0.00 |
| United Arab Emirates | 1 | 0.00 |
| **Total** | **47628** | **100** |

**Supplementary Table 2. Sample metadata distribution by body site and ruminant species (full table) ^1^**

| **Items** | **Sample counts** | **%** | **Ruminant species** |
| --- | --- | --- | --- |
| **Gut** | **30452** | **63.9** | C, S, G, Y, Bu, BC, DC, A, LL and Bi |
| Oesophageal | 5 |  | C |
| Rumen | 26652 |  | C, S, G, Y, Bu, BC, DC, A, LL and Bi |
| Reticulum | 131 |  | C, S, G, Y and Bu |
| Omasum | 150 |  | C, S, G, Y and Bu |
| Abomasum | 252 |  | C, S, G, Y, Bu and BC |
| Duodenum | 374 |  | C, S, G, Y, Bu and A |
| Jejunum | 567 |  | C, S, G, Y, Bu and A |
| Ileum | 496 |  | C, S, G, Y, Bu, BC and A |
| Cecum | 405 |  | C, S, G, Y, Bu and A |
| Colon | 658 |  | C, S, G, Y, and Bu |
| Rectum | 525 |  | C, S, G, Y, Bu and DC |
| Anus | 73 |  | S and G |
| Gut^2^ | 164 |  | C, BC, and DC |
| **Feces** | **10825** | **22.7** | C, S, G, Y, Bu, BC, DC, A, LL and Bi |
| **Respiratory system** | **1759** | **3.7** | C, S, Y and DC |
| Nasal | 1589 |  | C, S and Y |
| Larynx | 39 |  | C |
| Trachea | 41 |  | C |
| Lung | 90 |  | C and DC |
| **Milk** | **1389** | **2.9** | C, S and Bu |
| Milk | 1209 |  | C, S and Bu |
| Colostrum | 180 |  | C and S |
| **Fetal tissue** | **1001** | **2.1** | C and S |
| Umbilical cord | 6 |  | C |
| Placenta | 206 |  | C |
| Amniotic fluid | 75 |  | C |
| Allantoic fluid | 33 |  | C |
| Kidney | 203 |  | C |
| Liver | 210 |  | C |
| Rumen | 32 |  | C |
| Fetal gut | 28 |  | S |
| Ileum | 107 |  | S |
| Cecum | 52 |  | C and S |
| Meconium | 49 |  | C |
| **Skin** | **752** | **1.6** | C and S |
| Skin | 577 |  | C |
| Foot | 117 |  | S |
| Udder skin | 52 |  | C and S |
| Ventral skin | 6 |  | S |
| **Reproductive system** | **624** | **1.3** | C and S |
| Uterus | 337 |  | C and S |
| Vagina | 200 |  | C and S |
| Penis | 87 |  | C |
| **Oral** | **443** | **0.9** | C and S |
| Oral | 369 |  | C and S |
| Tonsil | 39 |  | C |
| Saliva | 35 |  | C |
| **Liver** | **133** | **0.3** | C |
| **Mammary gland** | **85** | **0.2** | C |
| Udder | 54 |  | C |
| Teat | 31 |  | C |
| **Blood** | **72** | **0.2** | C |
| **Eye** | **48** | **0.1** | C |
| **Musculoskeletal system** | **44** | **0.1** | C |
| Muscle | 31 |  | C |
| Joint | 13 |  | C |
| **Ears** | **1** | **0.0** | S |

^1^ C= Cattle, S= Sheep, G= Goat, Y= Yak, Bu= Buffalo, BC= Bactrian camel, DC= Dromedary camel, A = Alpaca, LL= Llama, and Bi= Bison.

^2^ Sample metadata tagged as gut.

**Supplementary Table 3. Sample metadata distribution from three major ruminant species by body site**

| **Items** | **Cattle** | | **Sheep** | | **Goat** | |
| --- | --- | --- | --- | --- | --- | --- |
|  | **Sample counts** | **%** | **Sample counts** | **%** | **Sample counts** | **%** |
| Blood | 72 | 0.2 | 0 | 0.0 | 0 | 0.0 |
| Eye | 48 | 0.1 | 0 | 0.0 | 0 | 0.0 |
| Feces | 7977 | 23.5 | 1214 | 13.5 | 421 | 22.8 |
| Fetal tissue | 850 | 2.5 | 151 | 1.7 | 0 | 0.0 |
| Gut | 20437 | 60.2 | 6966 | 77.4 | 1425 | 77.2 |
| Liver | 133 | 0.4 | 0 | 0.0 | 0 | 0.0 |
| Mammary gland | 85 | 0.3 | 0 | 0.0 | 0 | 0.0 |
| Milk | 1340 | 4.0 | 43 | 0.5 | 0 | 0.0 |
| Musculoskeletal system | 44 | 0.1 | 0 | 0.0 | 0 | 0.0 |
| Oral | 117 | 0.3 | 326 | 3.6 | 0 | 0.0 |
| Reproductive system | 617 | 1.8 | 7 | 0.1 | 0 | 0.0 |
| Respiratory system | 1614 | 4.8 | 130 | 1.4 | 0 | 0.0 |
| Skin | 587 | 1.7 | 165 | 1.8 | 0 | 0.0 |
| Ears | 0 | 0.0 | 1 | 0.0 | 0 | 0.0 |
| **Total** | **33921** | **100** | **9003** | **100** | **1846** | **100** |

**Supplementary Table 4. Goats sample metadata distribution by breed**

| **Items** | **Sample counts** | **%** |
| --- | --- | --- |
| Missing information | 921 | 49.9 |
| Liuyang black | 115 | 6.2 |
| Boer | 111 | 6.0 |
| Black Fattening | 86 | 4.7 |
| Xiangdong black | 80 | 4.3 |
| Cashmere | 76 | 4.1 |
| Guanzhong | 72 | 3.9 |
| Saanen | 48 | 2.6 |
| Murciano-Granadina | 32 | 1.7 |
| Guangxi | 30 | 1.6 |
| Hainan | 30 | 1.6 |
| Sichuan | 30 | 1.6 |
| Yunnan | 30 | 1.6 |
| Albas | 24 | 1.3 |
| Chuanzhong black | 24 | 1.3 |
| Qianbei-pockmarked | 18 | 1.0 |
| Lezhi black | 15 | 0.8 |
| Boer × Yangtze | 13 | 0.7 |
| Jianyang Daer | 13 | 0.7 |
| Japanese | 12 | 0.7 |
| Saanen | 12 | 0.7 |
| Ganxi Black | 11 | 0.6 |
| Tibetan | 8 | 0.4 |
| Black Bengal | 6 | 0.3 |
| Malabari | 6 | 0.3 |
| Osmanabadi | 6 | 0.3 |
| Salem Black | 6 | 0.3 |
| Guanzhong | 5 | 0.3 |
| Beshi | 3 | 0.2 |
| Harei | 3 | 0.2 |
| **Total** | **1846** | **100.0** |

**Supplementary Table 5. Goats sample metadata distribution by country**

| **Items** | **Sample counts** | **%** |
| --- | --- | --- |
| China | 1660 | 89.9 |
| Spain | 68 | 3.7 |
| India | 32 | 1.7 |
| Italy | 17 | 0.9 |
| Nigeria | 14 | 0.8 |
| Japan | 12 | 0.7 |
| France | 8 | 0.4 |
| USA | 7 | 0.4 |
| Saudi Arabia | 6 | 0.3 |
| Thailand | 6 | 0.3 |
| Argentina | 4 | 0.2 |
| Iran | 4 | 0.2 |
| Denmark | 2 | 0.1 |
| Mexico | 2 | 0.1 |
| Cuba | 1 | 0.1 |
| Hungary | 1 | 0.1 |
| Israel | 1 | 0.1 |
| Kenya | 1 | 0.1 |
| **Total** | **1846** | **100.0** |

**Supplementary Table 6. Goats sample metadata distribution by age**

| **Items** | **Sample counts** | **%** |
| --- | --- | --- |
| Missing information | 840 | 45.5 |
| Kid | 525 | 28.4 |
| Adult | 253 | 13.7 |
| Yearlings | 222 | 12.0 |
| Fetus | 6 | 0.3 |
| **Total** | **1846** | **100.0** |

**Supplementary Table 7. Goats sample metadata distribution by sex**

| **Items** | **Sample counts** | **%** |
| --- | --- | --- |
| Missing information | 1251 | 67.8 |
| Female | 481 | 26.1 |
| Male | 114 | 6.2 |
| **Total** | **1846** | **100.0** |

**Supplementary Table 8. Sample metadata distribution from minor ruminant species by body site and ruminant species**

| **Items** | **Sample counts** | **%** | **Ruminant species** |
| --- | --- | --- | --- |
| Gut | 1624 | 56.8 | Y, Bu, BC, DC, A, LL and Bi |
| Feces | 1213 | 42.4 | Y, Bu, BC, DC, A, LL and Bi |
| Respiratory system | 15 | 0.5 | Y and DC |
| Milk | 6 | 0.2 | Bu |
| **Total** | **2858** | **100** |  |

Y= Yak, Bu= Buffalo, BC= Bactrian camel, DC= Dromedary camel, A = Alpaca, LL= Llama, and Bi= Bison.

**Supplementary Table 9. Sample metadata distribution from minor ruminant species by country**

| **Items** | **Sample counts** |
| --- | --- |
| **Yak (*Bos grunniens*)** | **1280** |
| China | 1280 |
| **Buffalo (*Bubalus bubalis*)** | **996** |
| China | 810 |
| India | 106 |
| Thailand | 41 |
| Myanmar | 31 |
| New Zealand | 3 |
| Pakistan | 3 |
| Colombia | 2 |
| **Dromedary camel (*Camelus dromedarius*)** | **314** |
| India | 151 |
| Egypt | 108 |
| Iran | 44 |
| Saudi Arabia | 6 |
| USA | 2 |
| France | 2 |
| United Arab Emirates | 1 |
| **Alpaca (*Vicugna pacos*)** | **123** |
| USA | 115 |
| New Zealand | 8 |
| **Bactrian camel (*Camelus bactrianus*)** | **79** |
| Russia | 55 |
| China | 21 |
| Italy | 2 |
| Denmark | 1 |
| **Bison (*Bison bison*)** | **58** |
| USA | 38 |
| Canada | 19 |
| Mexico | 1 |
| **Llama (*Lama glama*)** | **8** |
| Argentina | 6 |
| France | 2 |
