# Supplementary figures and images for: Ruminant microbiome data are skewed and unFAIR, undermining their usefulness for sustainable production improvement
Source: Anim Microbiome. 2024 Oct 25;6:61. doi: 10.1186/s42523-024-00348-x (PMC11515148; doi:10.1186/s42523-024-00348-x)

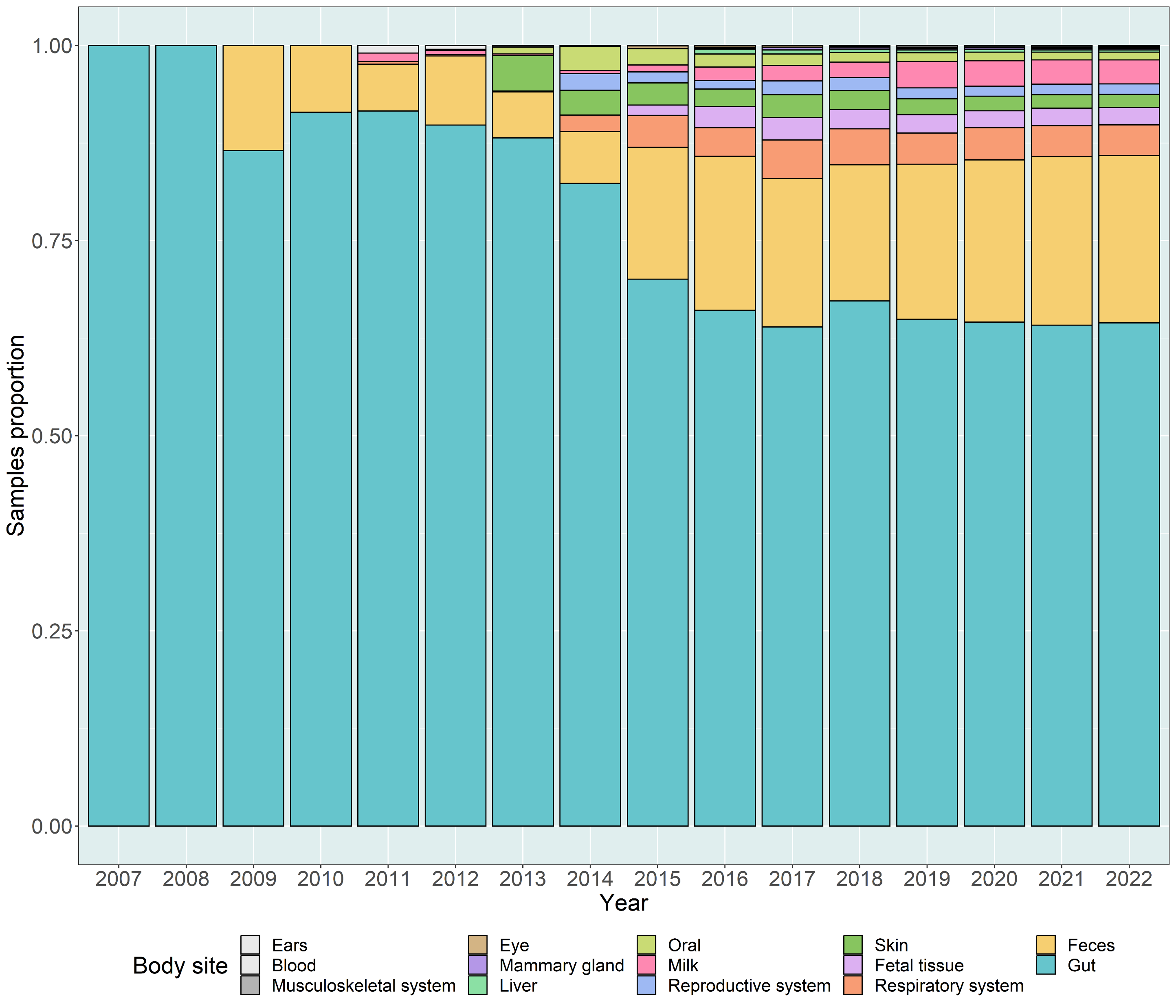

Supplement: Supplementary file 4 — Supplementary Material 4 [file 42523_2024_348_MOESM4_ESM.tif]
